# The Value of Contrast-Enhanced Ultrasound (CEUS) in the Detection of Perfusion Disturbances in Abdominal Wall Hernias Compared with Surgical and Histological Assessment

**DOI:** 10.3390/diagnostics12020370

**Published:** 2022-02-01

**Authors:** Ehsan Safai Zadeh, Christian Görg, Phillip Kuttkat, Christoph Frank Dietrich, Christina Carolin Westhoff, Fiona Rodepeter, Corinna Trenker, Marvin Görg, Andreas Kirschbaum, Amjad Alhyari

**Affiliations:** 1 Interdisciplinary Center of Ultrasound Diagnostics, University Hospital Giessen and Marburg, Philipps University Marburg, Baldingerstraße, 35033 Marburg, Germany; ehsan_sz@yahoo.de (E.S.Z.); p.kuttkat@ymail.com (P.K.); amjadalhyari@gmail.com (A.A.); 2 Gastroenterology, Endocrinology, Metabolism and Clinical Infectiology, University Hospital Giessen and Marburg, Philipp University of Marburg, Baldingerstraße, 35033 Marburg, Germany; 3 Department Allgemeine Innere Medizin (DAIM), Kliniken Hirslanden Bern, Beau Site, Salem und Permanence, 3018 Bern, Switzerland; c.f.dietrich@googlemail.com; 4 Institute of Pathology, University Hospital Giessen and Marburg, Philipps University Marburg, Baldingerstraße, 35033 Marburg, Germany; westhoff@med.uni-marburg.de (C.C.W.); rodepete@med.uni-marburg.de (F.R.); marvin.goerg@uk-gm.de (M.G.); 5 Haematology, Oncology and Immunology, University Hospital Giessen and Marburg, Philipps University Marburg, Baldingerstraße, 35033 Marburg, Germany; trenker@med.uni-marburg.de; 6 Department of Visceral, Thoracic and Vascular Surgery, University Hospital Giessen and Marburg, Philipps University Marburg, Baldingerstraße, 35033 Marburg, Germany; akirschb@med.uni-marburg.de

**Keywords:** ultrasound, CEUS, abdominal wall hernias, surgery, diagnosis

## Abstract

**Purpose:** This study aimed to evaluate the value of contrast-enhanced ultrasound (CEUS) in the evaluation of perfusion disturbance in irreducible abdominal wall hernias (AWHs). **Methods:** From 2006 to 2018, 50 patients with an irreducible AWH were examined using B-mode ultrasound (B-US) and CEUS. The ultrasound findings were correlated with subsequent surgical and histological results. The presence of non-enhanced areas (NEAs) in hernia contents on CEUS and the presence of non-perfused areas (NPAs) on surgical and histological evaluation were analyzed retrospectively. **Results:** On CEUS, 13/50 hernia contents (26.0%) revealed NEAs during complete CEUS examination and 37/50 (74.0%) revealed no NEAs during CEUS examination. On surgical and histological evaluation, NPAs in hernia contents were identified in 11/13 cases (93.3%) with NEAs on CEUS. CEUS was found to have a sensitivity of 100.0%, a specificity of 94.9%, a positive predictive value of 84.6%, and a negative predictive value of 100.0% for the identification of perfusion disturbance in AWHs. **Conclusions:** The findings of this study demonstrate that using CEUS as an imaging method may be helpful for evaluating the perfusion of hernia contents in incarcerated AWHs. On CEUS, the presence of NEAs may suggest perfusion disturbance in hernia contents.

## 1. Introduction

Abdominal wall hernia (AWH) is a common disease. Its prevalence depends on age and is described in the literature to be 1.7% for all ages and 4.0% for people over 45 years old [1,2,3]. Furthermore, it is estimated that more than 20 million hernias are operated on worldwide each year [4]. Although surgical correction is elective in most patients, approximately 5.0–13.0% of patients require emergency surgery due to a strangulated AWH with blood flow disturbance in the hernia contents (omentum or bowel) [5]. Emergency cases are associated with high morbidity ranging from 19% to 30% (e.g., pulmonary and cardiac complications, anastomotic leakage and ileus, and wound infections) and high early mortality (within 30 days after an operation or before discharge from the hospital) ranging from 1.6% to 19.4% [6]. The most important prognostic factor in these patients is the time from the onset of symptoms to surgical therapy [7]. Therefore, the early detection of perfusion disturbance due to a strangulated AWH may be beneficial for identifying patients who require urgent surgery and could improve the outcome of these patients [7].

A widely used and proven imaging modality in clinical practice for the detection of perfusion disturbances is contrast-enhanced ultrasound (CEUS) [8,9,10,11,12].

The aim of this study was to determine the diagnostic value of CEUS in the detection or exclusion of perfusion disturbance in the hernia contents in irreducible AWH subsequently managed surgically.

## 2. Materials and Methods

From February 2006 to April 2018, 75 patients with a clinical irreducible AWH were examined in a university ultrasound (US) center. All the patients were investigated and standardized using B-mode ultrasound (B-US) and CEUS by a German Society for Ultrasound in Medicine (DEGUM) Level III-qualified examiner (C.G., internal medicine) using CEUS [13]. The inclusion criteria for the retrospective analysis were (1) confirmation of the diagnosis by surgical intervention and (2) surgical evaluation and histopathological examination of the hernia contents regarding disturbed perfusion.

In total, 50 patients were included in the study, and informed consent was obtained from each patient for the US examinations. This retrospective study was approved by the local ethics committee and conducted in accordance with the amended Helsinki Declaration on the ethical principles for medical research involving human subjects.

The US examinations were carried out with an ACUSON SEQUOIA 512 GI US scanner (Siemens, Erlangen, Germany) and a 4C1 curved-array transducer. For the subsequent CEUS examination of hernia contents, a frequency of 1.5 MHz was used in contrast-specific mode. The CEUS investigations were performed according to the European Federation of Societies for Ultrasound in Medicine and Biology (EFSUMB) guidelines [8]. A bolus injection of 2.4 mL of SonoVue contrast medium (Bracco Imaging S.p.A., Milan, Italy) was administered via peripheral venous access, followed by 10 mL of 0.9% NaCl. The hernia contents were continuously examined for the first 30 s. Subsequently, several short examinations were performed at 30 s intervals up to 2 min, and the changes in the perfusion pattern were saved as images. The following data were retrospectively evaluated.

### 2.1. Ultrasound Data

**Figure 1:**
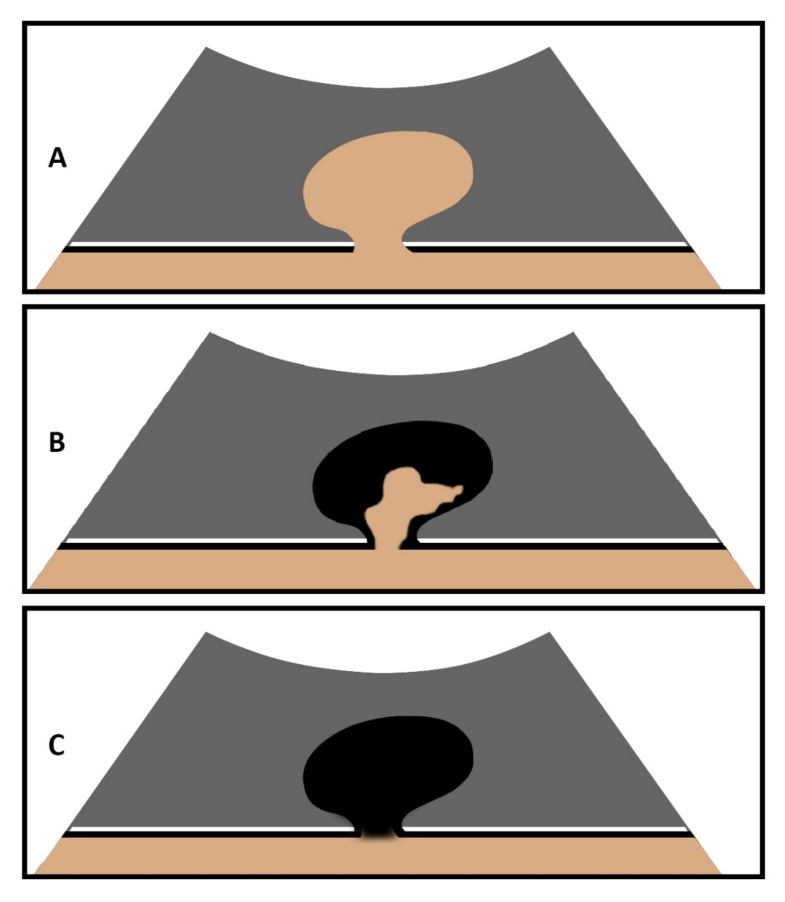
Graphical illustration of an abdominal wall hernia on CEUS with marked contrast enhancement of hernia content (**A**), partial absent enhancement of hernia content (**B**), and complete absent enhancement of hernia content (**C**).

1. The hernia contents were classified as omentum or bowel by B-US. Hernia contents with an echogenic mass and without detection of bowel loops were defined as an omental hernia, and the clear detection of bowel loops with mixed echogenicity due to a reverberation artifact of air in the bowel loops was defined as an intestinal hernia [14,15]. The simultaneous presence of bowel and omentum was considered an intestinal hernia.
2. The presence of non-enhanced areas (NEAs) in hernia contents (Figure 1B,C) was assessed during the CEUS examination [8,10].

The US data were retrospectively analyzed by two independent, experienced investigators (E.S. and C.G.). In the event of discrepancies, the final decision was made by a third experienced investigator (A.A.). The investigators were blinded to the surgical and histopathologic results during the evaluation of the US data.

### 2.2. Surgical and Histopathological Evaluation

1. Hernia contents were evaluated during surgical intervention and classified as omentum, bowel, or an empty sac.
2. Macroscopic and microscopic confirmation or exclusion of non-perfused areas (NPAs) (necrosis, hemorrhage, and fibrosis) in hernia contents was conducted.

All resected tissues were subjected to hematoxylin–eosin staining, and all tissue samples were evaluated microscopically by an experienced pathologist at the local Institute of Pathology.

### 2.3. Statistical Analysis

Sensitivity, specificity, and positive and negative predictive values were evaluated for pathological US findings. Cohen’s kappa statistics were applied to measure interrater reliability, and a *p*-value of <0.05 was defined as significant.

## 3. Results

### 3.1. Demographic and Clinical Data

Of the 50 participants, 25 were male and 25 were female. The mean age of the patients was 62.3 years (with a range from 21 to 91 years). The clinical data of the patients are shown in Table 1.

Surgical correction was performed within 24 h after the CEUS examination in 21/50 patients (42.0%), within 7 days after the CEUS examination in 14/50 patients (28.0%), and >7 days after CEUS examination in 15/50 patients (30.0%).

### 3.2. Ultrasound Data

**Figure 2:**
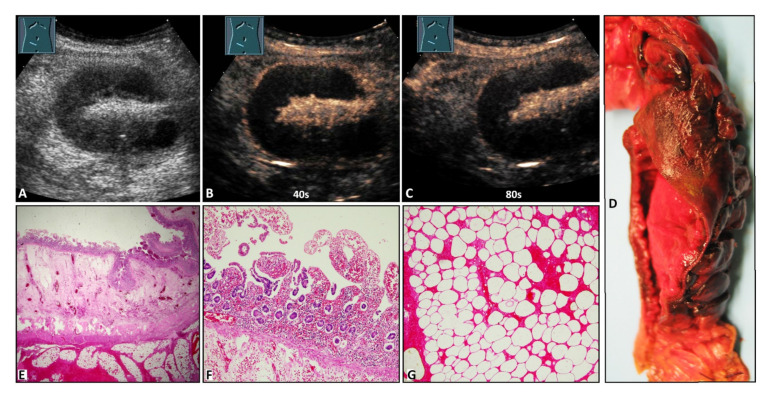
A 42-year-old male patient with acute peritoneal pain in the right lower abdomen and a palpable subcutaneously located tumor in the form of a Spieghel’s hernia. (**A**) B-mode ultrasound shows a standing loop of small bowel. (**B**,**C**) Contrast-enhanced ultrasound shows absent enhancement of the bowel wall after 40 s and 80 s. (**D**) Macroscopic evaluation shows a color change to deep red in the mucosa in an 8 cm long segment. (**E**) Cross section of the small intestine with luminal mucosa, edematous submucosa, muscularis, and hemorrhagic submucosa (from top to bottom). The mucosa shows incomplete ischemic necrosis with vital crypt epithelium (1.25× magnification). (**F**) Small intestinal mucosa with incomplete ischemic necrosis with vital crypt epithelium (10× magnification). (**G**) Necrotic mesenteric fat tissue with missing nuclei and hemorrhages (10× magnification).

**Figure 3:**
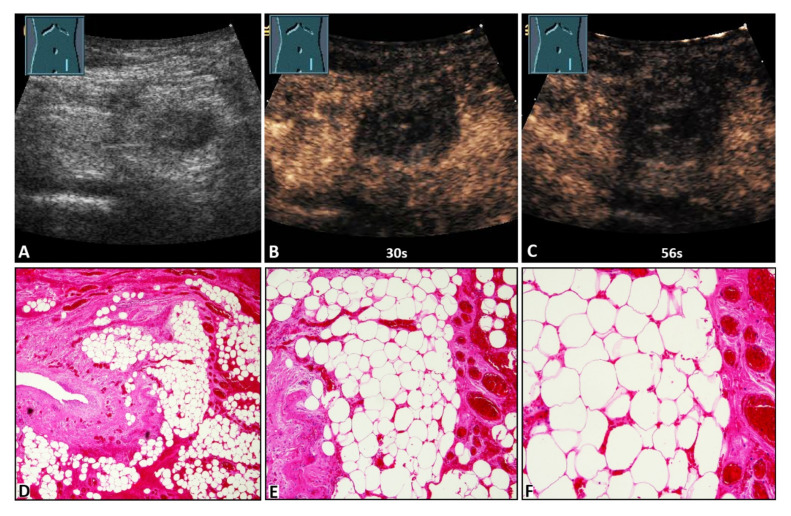
A 47-year-old female patient with acute peritoneal pain in the left lower abdomen and a palpable inguinal-located tumor. (**A**) B-mode ultrasound shows a hyperechoic lesion with a central hypoechoic area. (**B**,**C**) On contrast-enhanced ultrasound, the lesion shows absent enhancement after 30 s and 56 s, and an omental hernia was diagnosed. (**D**) Omentum with partial fibrotic consolidation (on the left) and dilated hyperemic capillaries and hemorrhages (4× magnification). (**E**) Omentum with central necrotic fat tissue (missing nuclei), fibrosis (to the left), and dilated hyperemic capillaries and hemorrhages (on the right) (10× magnification). (**F**) Omentum with central necrotic fat tissue, and dilated hyperemic capillaries and hemorrhages (20× magnification).

**Figure 4:**
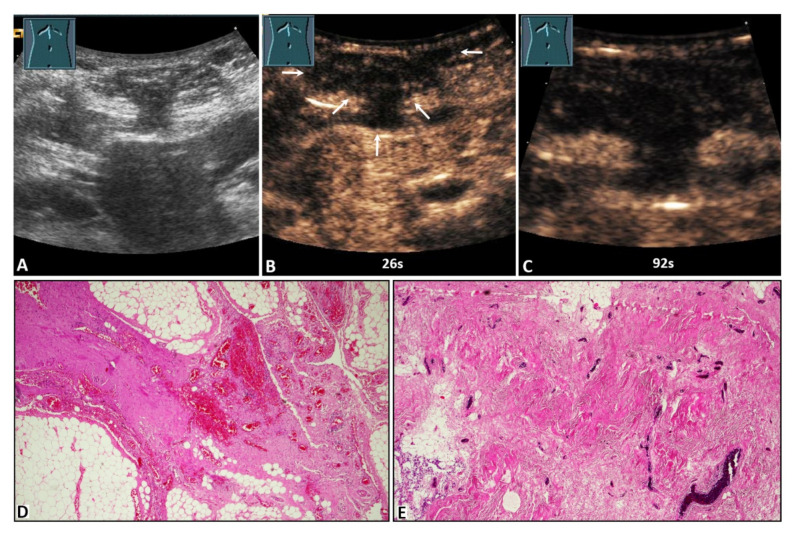
A 59-year-old male patient with acute peritoneal pain in the umbilical region and a palpable tumor. (**A**) B-mode ultrasound shows a complex lesion located in the abdominal wall, with visualization of omental hernia content. (**B**,**C**) On contrast-enhanced ultrasound, the lesion shows absent enhancement after 26 s (arrows) and 92 s. (**D**) Omentum with fibrotic strands, including dilated hyperemic capillaries (4× magnification). (**E**) Fibrotic hernia sac with dilated hyperemic capillaries (10× magnification).

**Figure 5:**
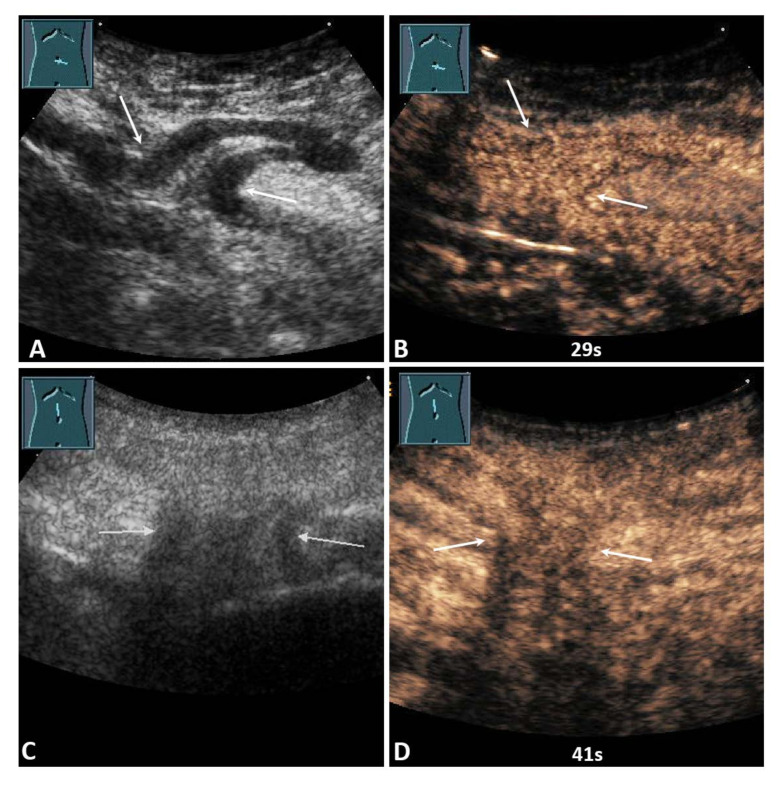
(**A**) A 59-year-old male patient with acute pain in the paraumbilical region. B-mode ultrasound shows a hernia orifice (arrows), and the hernia sac shows a fixed bowel structure. (**B**) On contrast-enhanced ultrasound, the loop of small bowel shows a marked enhancement after 29 s. Surgical evaluation showed no evidence of infarction. No resection of the bowel was performed. (**C**) A 91-year-old female patient with acute pain in the epigastric region. B-mode ultrasound shows a hernia orifice (arrows), and the hernia sac shows fixed omental tissue. (**D**) On contrast-enhanced ultrasound, the hernia contents show a marked enhancement after 41 s. Surgical evaluation showed no evidence of infarction. No resection of the omentum was performed.

On B-US, the hernia contents were identified as omentum in 27/50 cases (54.0%) and as small bowel in 23/50 cases (46.0%). On CEUS, 13/50 hernia contents (26.0%) revealed NEAs during the complete CEUS examination (Figure 2, Figure 3 and Figure 4), whereas 37/50 hernia contents (74.0%) revealed no NEAs during the complete CEUS examination (Figure 5).

The agreement between the examiners for the US finding was good (Cohen’s kappa = 0.79).

### 3.3. Surgical Evaluation, Histopathological Examination, and Their Correlation with Ultrasound Data

Surgical correction was performed in all patients. Macroscopic evaluation during the surgical intervention and/or histological examination of the resected tissue showed the hernia contents to be omentum in 25/50 cases (50.0%) and bowel in 20/50 cases (40.0%). In 5/50 cases (10.0%), the hernia sac was empty. In 2/50 cases (4.0%), the hernia contents were demonstrated by the US to be bowel and in the surgical evaluation to be omentum (Table 2). The hernia contents were consistent with the US findings in 44/50 cases (88.0%) (Cohen’s kappa = 0.78, ‘good’).

In 32/50 cases (64.0%), no resection of the hernia contents was performed. In the remaining 18/50 cases (36.0%), a resection of the omentum (13/50; 26.0%) and of the bowel (5/50; 10.0%) was performed.

On histological examination of the resected tissue samples, NPAs were absent in 7/18 cases (38.9%). NPAs (necrosis, hemorrhage, and fibrosis) were present in 11/18 cases (61.1%), including bowel infarction (4/11), omental infarction (3/11), and omental fibrosis (4/11).

With regard to the correlation of NEAs to NPAs, of the 13 patients with NEAs on CEUS, NPAs were detected on histopathologic examination of 11/13 cases (93.3%) (Table 3). In the remaining two patients with NEAs on CEUS with omental hernias, emergency surgical correction was immediately performed (<6 h after the CEUS examination). CEUS was found to have a sensitivity of 100.0%, a specificity of 94.9%, a positive predictive value of 84.6%, and a negative predictive value of 100.0% for the identification of perfusion disturbance in the AWH contents.

## 4. Discussion

The World Society of Emergency Surgery guidelines recommend contrast-enhanced computed tomography (CT) as the imaging modality of choice for diagnosing strangulated AWHs [16]. In addition to contrast-enhanced CT, B-US is widely used for the evaluation of strangulated AWHs as the initial imaging modality and the expanded form of physical examination [17,18]. However, to the best of our knowledge, no data are available regarding the diagnostic performance of CEUS as an additional imaging modality in the diagnosis of perfusion disturbance in strangulated AWHs. In a standardized and surgically controlled study, we investigated the diagnostic accuracy of B-US for the evaluation of the hernia contents and the diagnostic accuracy of CEUS in the detection of perfusion disturbance in the hernia contents of 50 patients with an irreducible AWH.

Regarding the hernia contents, the US findings agreed with the surgical evaluations in 44/50 cases (88.0%). The difference in the remaining six cases could be due to the spontaneous reduction in hernia contents. It has been described previously in the literature that an incarcerated hernia can reduce spontaneously or during the administration of muscle relaxants during induction of anesthesia [19,20].

Regarding perfusion disturbance in the hernia contents, CEUS revealed a sensitivity of 100.0%, a specificity of 94.9%, a positive predictive value of 84.6%, and a negative predictive value of 100.0% for the identification of histologically proven perfusion disturbance of the AWH contents. In the two patients with NEAs on CEUS and no evidence of perfusion disturbance on histological examination, emergency surgical corrections were performed within 6 h after the CEUS examination. Therefore, it could be speculated that tissue damage would not have occurred in these two patients due to the short time between incarceration and surgical intervention with the successful restoration of reperfusion. The sensitivity and specificity of CEUS in the present study compared with the findings of previously performed color Doppler sonography and contrast-enhanced CT studies are summarized in Table 4.

The reason for the higher sensitivity of CEUS compared to a contrast-enhanced CT may be the strictly intravascular characteristics of the contrast medium of CEUS [22]; therefore, the non-perfused areas show an absence of enhancement during the complete CEUS examination, distinguished from perfused tissue. However, omental hernias were included in the present study. Notably, in a study by Jancelewicz et al., only the diagnostic performance of CECT in detecting a strangulated small bowel was investigated [21]. Therefore, a comparison between both studies is limited.

The high sensitivity and specificity of CEUS in the diagnosis of perfusion disturbance in incarcerated hernias demonstrate that CEUS should be considered a useful and cost-effective method without a radiation-exposing imaging modality. The findings of this study are in accordance with previous studies that showed that CEUS has a high diagnostic performance in the detection of perfusion disturbance [8,9,23,24,25,26,27,28,29]. Notably, the cause of NEAs in CEUS was an omental tissue fibrosis in four cases. This finding demonstrates that, in omental hernias, omental tissue fibrosis should be considered a differential diagnosis in addition to an acute perfusion disturbance. The fibrosis could be the result of recurrent incarcerations with chronic impaired perfusion and hypoxia [30].

There were limitations to this study: The sample size was relatively small. Furthermore, this study was limited by the retrospective data collection and, generally, by the well-known high interobserver variability of the US. Moreover, blinding of the investigators to the study group and blind interpretation of the US data by the US examiners were not possible. The study was performed on patients who were referred to the Interdisciplinary Centre of Ultrasound Diagnostics for the investigation of an irreducible AWH and were examined and standardized by a single DEGUM Level III-qualified examiner. Therefore, it was not possible to exclude selection bias. Another limitation of our study is the semiquantitative analysis of the US data, which probably allows more room for interpretation than a quantitative measurement, although interrater observer variability for the US findings was performed with good agreement.

## 5. Conclusions

The findings of this study demonstrate that the presence of NEAs on CEUS may be suggestive of perfusion disturbance. Furthermore, the CEUS pattern of omental infarction was described as a rare disease, and it was shown that omental fibrosis should be considered as a differential diagnosis in addition to acute infarction in the event of the presence of NEAs in the omentum. In summary, the findings of this study demonstrate that using CEUS as an imaging method may be helpful for evaluating the perfusion of hernia contents in incarcerated AWHs. The potential of CEUS in the clinical diagnostic algorithm of irreducible AWHs should be evaluated in a prospective randomized study.

## Figures and Tables

**Table 1:** Distribution of abdominal hernias in N = 50 study patients.

Clinical Diagnosis	Number (%)
Inguinal hernia	17 (34.0)
Incisional hernia	15 (30.0)
Epigastric hernia	9 (18.0)
Umbilical hernia	7 (14.0)
Femoral hernia	1 (2.0)
Spieghel’s hernia	1 (2.0)

**Table 2:** B-mode ultrasound data of abdominal hernia and its correlation with surgical/histopathological evaluation in terms of hernia contents.

Contents of Hernia Sac	Bowel in Surgery 20/50 (40.0%)	Omentum in Surgery 25/50 (50.0%)	Empty in Surgery 5/50 (10.0%)
Bowel in ultrasound 23/50 (46.0%)	20/23 (87.0%)	1/23 (4.3%)	2/23 (8.7%)
Omentum in ultrasound 27/50 (54.0%)	0/27 (0.0%)	24/27 (88.9%)	3/27 (11.1%)

**Table 3:** Contrast-enhanced ultrasound data of abdominal hernia and its correlation with surgical/histopathological evaluation.

Clinical Diagnosis	NPAs on Surgical/Histopathological Examination 11/50 (22.0%)	No NPAs on Surgical/Histopathological Examination 39/50 (78.0%)
NEAs on CEUS 13/50 (34.0%)	11/13 (84.6%)	2/13 (15.4%)
No NEAs on CEUS37/50 (74.0%)	0/37 (0.0%)	37/37 (100.0%)

CEUS: contrast-enhanced ultrasound; NEA: non-enhanced area; NPA: non-perfused area.

**Table 4:** Diagnostic performance of color Doppler sonography, contrast-enhanced ultrasound, and contrast-enhanced computed tomography for evaluating perfusion disturbance.

Imaging Modality	Cases	Year	Author	Sensitivity (%)	Specificity (%)
CDS	149	2001	Rettenbacher et al. [18]	22.0	75.0
CECT	192	2009	Jancelewicz et al. [21] *	56.0	94.0
CEUS	50	2021	Present study	100.0	94.6

CDS: color Doppler sonography; CECT: contrast-enhanced computed tomography; CEUS: contrast-enhanced ultrasound. * Diagnostic performance of CECT in the detection of strangulated small bowel obstruction.

## Data Availability

The data presented in this study are available from the corresponding author upon request.
